# Children’s vicarious ratings of social touch are tuned to the velocity but not the location of a caress

**DOI:** 10.1371/journal.pone.0256303

**Published:** 2021-08-26

**Authors:** Connor J. Haggarty, Paula D. Trotter, Francis McGlone, Susannah C. Walker

**Affiliations:** 1 Research Centre for Brain & Behaviour, School of Psychology, Liverpool John Moores University, Liverpool, United Kingdom; 2 Institute of Psychology, Health & Society, University of Liverpool, Liverpool, United Kingdom; University of Ottawa, CANADA

## Abstract

Affective sharing is a bottom-up process involving automatic processing of sensory inputs that facilitate vicarious experience of another’s emotional state. It is grounded directly in the prior experiences of the perceiver. In adults, vicarious ratings of affective touch match the known velocity tuning and hypothesised anatomical distribution of C-tactile afferents (CT), a subclass of C-fibre which respond preferentially to low force/velocity stroking touch, typically perceived as pleasant. Given the centrality of touch to early nurturing interactions, here we examined whether primary school aged children’s vicarious ratings of affective touch show the same anatomical and velocity specific patterns reported in adults. Forty-four children aged between 8 and 11 (mean age 9, 24 male) rated a sequence of video clips depicting one individual being touched by another on 5 different upper-body sites (palm, dorsal forearm, ventral forearm, upper-arm and back) at 3 different velocities (static, CT optimal, slow stroking and non-CT optimal, fast stroking). Immediately after viewing each clip, participants were asked to rate how pleasant they perceived the touch to be. While children rated the CT optimal velocity significantly higher than static or non-CT optimal touch, unlike adults their ratings did not vary across skin sites. This difference may reflect the fact children’s ratings are grounded in bottom-up affective resonance while adults also draw on top-down cognitive evaluation of the broader social context when rating the stimuli.

## 1. Introduction

Affective resonance is a bottom-up process which involves automatic processing of sensory inputs that facilitate vicarious experience of another’s emotional state [1]. The neural basis of affective sharing has been widely studied using pain observation paradigms, which reveal significant overlap between brain areas activated during vicarious and first-hand experience of pain [2–4]. Affective resonance for pain can be observed whether an actor’s facial expressions are being observed or not [4–6]. For example, viewing body parts in a painful condition in the absence of a broader social context results in common activation of brain regions as self-experienced pain [6]. The perception-action coupling mechanisms which underpin affect sharing are present implicitly from birth [7, 8] and become explicit with experience through childhood and adolescence [9, 10]. They are thus likely to provide the initial mechanism upon which, higher order, top-down processes required for cognitive empathy are built [1].

Just as with pain, people show affective empathy for vicariously experienced touch [11–14]. For example, viewing another person receive social, caressing touch elicits the same velocity dependent responses in the posterior insula cortex as are reported when the same stimulus is experienced first-hand [14]. These findings suggest that the velocity of touch is a key feature which people use to determine its affective content. Indeed, there is neurobiological support for this hypothesis; a class of unmyelinated c-fibres has been identified and characterised in the hairy skin of mammals that are tuned to exactly the stimulus velocities which, in psychophysical studies, participants perceive as most pleasant [15–17] That is, single unit microneurography recordings show these c-tactile afferents (CTs) fire most prominently to a stimulus moving across their receptive field at 1-10cm/s, the very velocity range which reliably produces the highest hedonic ratings when participants experience gentle stroking touch [16].

Evidence that affect sharing is grounded directly in the prior experiences of the perceiver comes from study of patients carrying a heritable mutation which leads to reduced c-fibre density [18]. In addition to blunted temperature and pain sensitivity, patients with this hereditary sensory and autonomic neuropathy type V (HSAN-V) mutation do not report velocity dependent ratings of gentle, moving touch and furthermore, show exactly the same flattened pattern of ratings to vicariously experienced touch. Neurally, whether experiencing touch first-hand or vicariously, HSAN-V patients’ responses within posterior insular cortex showed no distinction between stimuli moving at a CT optimal 3cm/s or a non-CT optimal 30cm/s. Overall, despite reporting the same levels of interpersonal touch as the control group, HSAN-V patients haven’t learned that stimulus velocity is an important cue for judging its affective value [18]. These data provide important evidence both that vicarious ratings are grounded in the viewer’s own perceptual experience and that CTs contribute to the development of pleasant touch perception. Further indication that personal experience shapes affective touch ratings comes from a recent psychophysical study where young adults, who as children experienced abuse and or neglect, showed blunted sensitivity to CT-targeted touch, whether experienced first-hand or vicariously. Again, this was despite the fact they reported the same levels of current intimate social touch as the control group [19].

The notion that early social tactile experience shapes adult perceptions of affective touch is not surprising given the centrality of touch to early nurturing interactions and its role in promoting attachment formation [20–22 for reviews] Indeed, neuroimaging data shows that the CT system is functional from birth [23, 24] and parents spontaneously caress their infant at CT optimal velocities [25–27]. While neural differentiation between discriminative and affective aspects of touch develops over the first 12 months of life [28–31], behaviourally, by 9 months of age, children show an attentional bias to CT-targeted over faster or slower skin stroking [32]. Indeed, once old enough to use a rating scale, in a psychophysical study young children showed similar, velocity dependent ratings of affective touch as adults [33].

In addition to velocity, another cue to the affective value of social touch is location. While the anatomical distribution of CTs in human skin is not known, psychophysical studies have reported variation in the perceived pleasantness of CT-targeted touch across skin sites [15, 34, 35] and biopsy studies reported higher density of epidermal c-fibres on the back than proximal limb sites [36]. This is consistent with molecular genetic visualization of massage responsive C-low threshold mechanoreceptors (CLTMs–the presumed rodent homologue of CTs) in mice which revealed a denser distribution in dorsal than ventral thoracic sites, greater proximal than distal limb innervation and a complete absence from glabrous paw skin [37]. Though velocity tuning of rodent CLTMs has not been established, stroking touch applied to rats at CT optimal velocities elicits dopamine release within the nucleus accumbens (NAC). The effect is anatomically specific, as stroking applied to the back elicited a significantly greater dopamine response than stroking the limbs or abdomen [38]. Consistent with this report, several behavioural studies, in humans and animals, have established that selective CLTM activation, or application of touch which should optimally activate CTs, is motivating and its rewarding value is learned rapidly [39–42].

We have previously reported that adults’ affective responses to observed social touch reflect the predicted anatomical distribution and known velocity tuning of CTs [19, 43]. That is, we observed the same velocity dependent psychophysical response curves in ratings of observed touch, delivered on CT innervated hairy skin sites, as have been reported to felt touch. Furthermore, people rated touch on the back, where CT innervation is hypothesized to be most dense, higher than on more proximal sites. While, in psychophysical tests using directly felt touch, the same velocity dependent relationship between pleasantness and touch has been reported on the palm as on the arm [44], we did not see the equivalent velocity tuned profile in response to observed touch to the palm [19, 43]. This difference may reflect the fact our stimuli included a static touch condition, which while being highly relevant to many social tactile interactions, such as hand holding, is not generally studied psychophysically, where dynamic stroking is typically used.

The aim of the present study was to determine whether children’s ratings of vicariously experienced affective touch show the same velocity and anatomical specificity as adult ratings. We hypothesised, given the centrality of touch to nurturing interactions and early behavioural sensitivity to the specific rewarding value of CT targeted touch, that such preferences would be observable early in development and so children’s rating patterns would match those previously reported in adults.

## 2. Methods

### 2.1 Participants

Forty-four children aged 8–11 years (mean 9 years +/- 0.9, 24 male), were recruited from years 4, 5 and 6 of a primary school in the Northwest of England. Parents/Guardians gave written informed consent for their child’s participation. Each child also provided informed assent before beginning the study. The study was approved by the LJMU Psychology Research Ethics Committee.

### 2.2 Materials & methods

**Fig 1:**
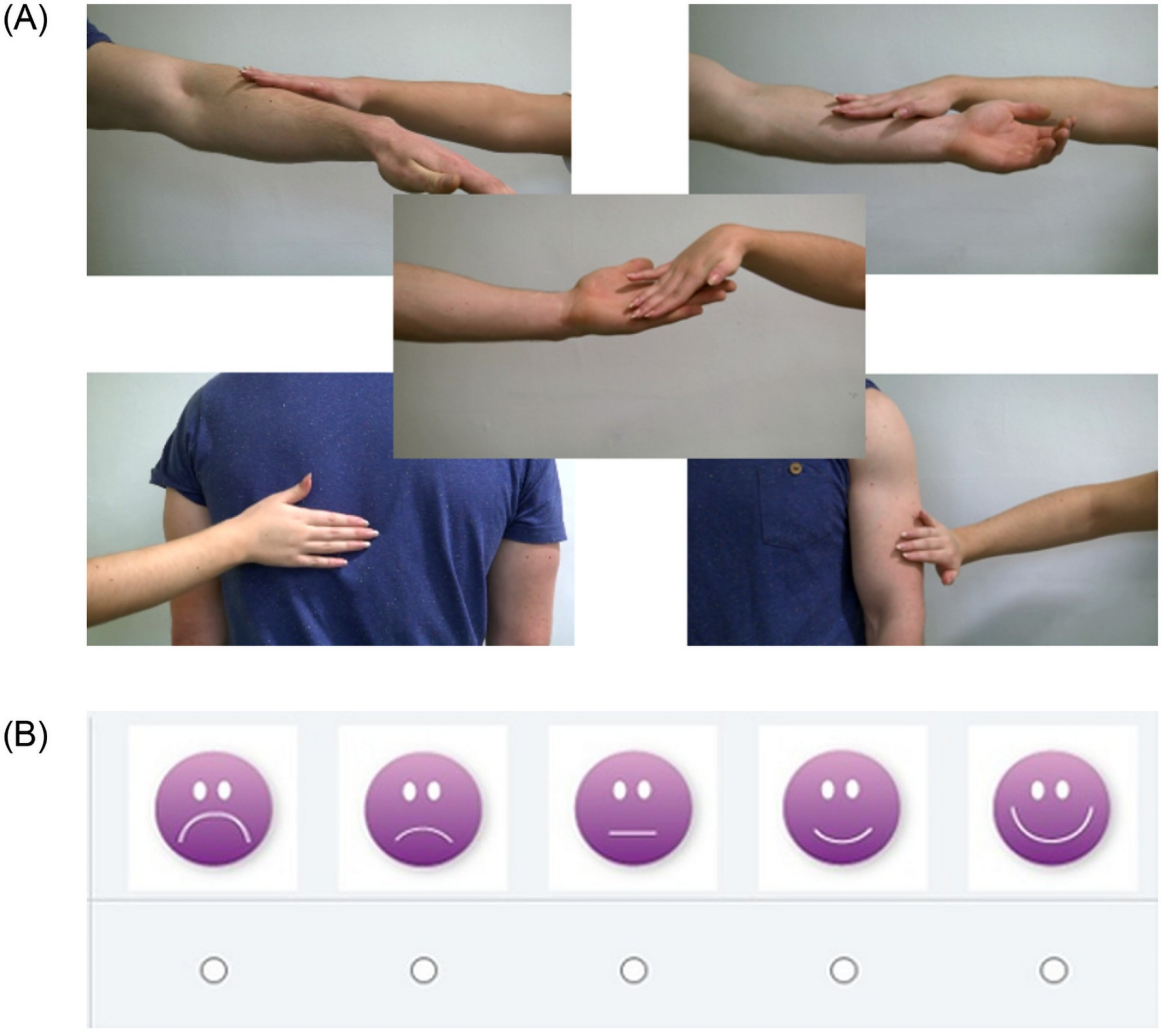
**(A)** Stills from the videos presented, one depicting each of the 5 locations studied. The clips lacked any social context, faces were not visible, and showed only the hand and forearm of one female actor “the toucher” and the relevant upper body part (back, arm or palm) or the other male actor “the receiver.” **(B)** Example of the smiley face scale used for measuring the children’s affective touch ratings. (adapted from 35,47).

Participants viewed and rated a random sequence of 15 short (5 sec) videos depicting one male individual being touched by a female at 5 different skin sites (back, upper arm, ventral forearm, dorsal forearm and palm) and at 3 different velocities (Static touch, slow—CT optimal strokes and fast–non-CT optimal strokes). (Fig 1A shows video stills, depicting the 5 body sites investigated) [43]. Immediately after viewing each clip, a new screen appeared where participants were asked make a hedonic rating using a smiley face scale (designed and validated for use with young children by Cascio et al & Croy et al [33, 45]—see Fig 1B): (1) Thinking about the video you have just watched answer the question below by choosing a face. How nice do you think it was for the person being touched? (2) Again, thinking about the same video answer the question below by choosing a face. How much would you like to be touched like that? These two questions always appeared in the same order, each on a new screen, with question 2 appearing directly after the response to question 1 was made. They were designed to probe expectations of how touch is perceived by others versus self.

### 2.3 Procedure

Children completed the experiment in a computer classroom at school. All testing took place on the same morning and was conducted in 4 sessions with 8–13 children present in each. Initially, the experimenter read through a short information sheet with the group and before beginning, children were asked if they still wished to take part. To prevent students being influenced by their peers they were asked to sit at computers with an empty space between each other. The experimenter then gave a brief overview of what they were going to do. To test their understanding of how to use the rating scale, initially several images of different types of food were displayed on a projector screen at the front of the room. Children were instructed to use the smiley face scale to rate the different types of food. The food shown included a variety of items that the children were likely to really like, items they were likely to rate neutrally and finally items they were likely to dislike (Images included, chocolates and French fries, carrots, beans, strawberries and apples, sprouts and mushrooms). Each type of food was displayed one at a time in a random order. Once it was clear to the experimenter that all the children understood how to use the scale, further instructions were given. Children were told that in a few moments, they would view a series of videos each showing a person being touched. It was made clear that two questions would appear following each video, which they were required to answer honestly as there were no right or wrong answers. Before the first video was shown, children were required to enter their age. If the students had no further questions, they were instructed to begin. The children watched each video at their own computer, allowing them to work through the questions at their own pace. After all the children had finished, they were fully debriefed and returned to their classes. The study was hosted in Qualtrics version 04 2018 (Provo, UT) survey software.

### 2.4 Statistical analysis

Following the procedure of Croy et al [33], in their previous use of this rating scale to measure children’s perception of affective touch, the ratings from the pictorial scales were converted into numbers (1-very bad, 2-bad, 3-neutral, 4-happy, 5-very happy). Subsequently, data were analyzed in SPSS (Version 26) using a generalized linear model with ordinal logistic link function; Velocity (3 levels) and Location (5 levels) were entered as within subject factors, subject was entered as a random factor. Significant main effects were followed up using Wilcoxon Signed Ranks tests for non-parametric data. Due to a coding error, it is not possible to match up a participant’s gender to their age and touch rating data. Figures were drawn using R packages tidyverse and ggsignif.

## 3. Results

### Q1. How nice do you think it was for the person being touched?

**Fig 2:**
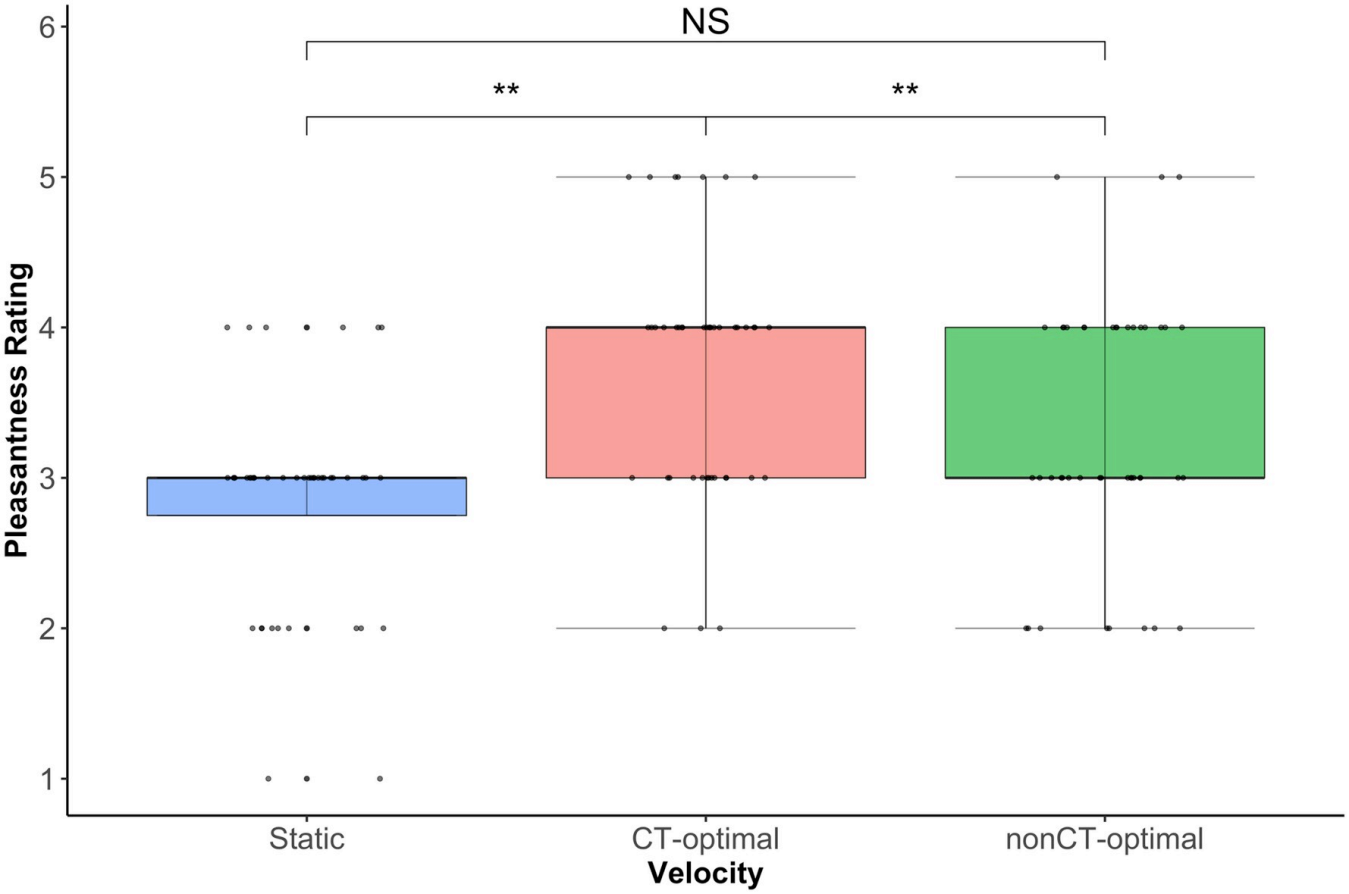
Box and whisker plot showing median pleasantness ratings (thick black line) for touch at the 3 stroking velocities for question 1. Dots represent individual participant ratings. There is a significant main effect of velocity (p < 0.001), but no effect of location, or location x velocity interaction. Wilcoxon Signed Ranks tests confirmed that the CT optimal velocity, slow stroking touch was rated significantly more positively than either static or fast stroking, non-CT optimal velocity touch, **ps < 0.002.

A significant main effect of velocity was identified (Wald χ^2^(2) = 34.11, *p* < 0.001), with Wilcoxon Signed Ranks tests confirming CT optimal (~3cm/s) touch was rated significantly more positively than the other two velocities (*p*s < 0.002). While there was a trend for static touch to be rated less positively than fast (~30cm/s) touch, this did not reach the threshold for significance (*p* = 0.06). There was no significant main effect of location (Wald χ^2^(4) = 2.33, *p* = 0.68), nor was there a significant location by velocity interaction (Wald χ^2^(8) = 14.28, *p* = 0.08). See Fig 2.

### Q2. How much would you like to be touched like that?

**Fig 3:**
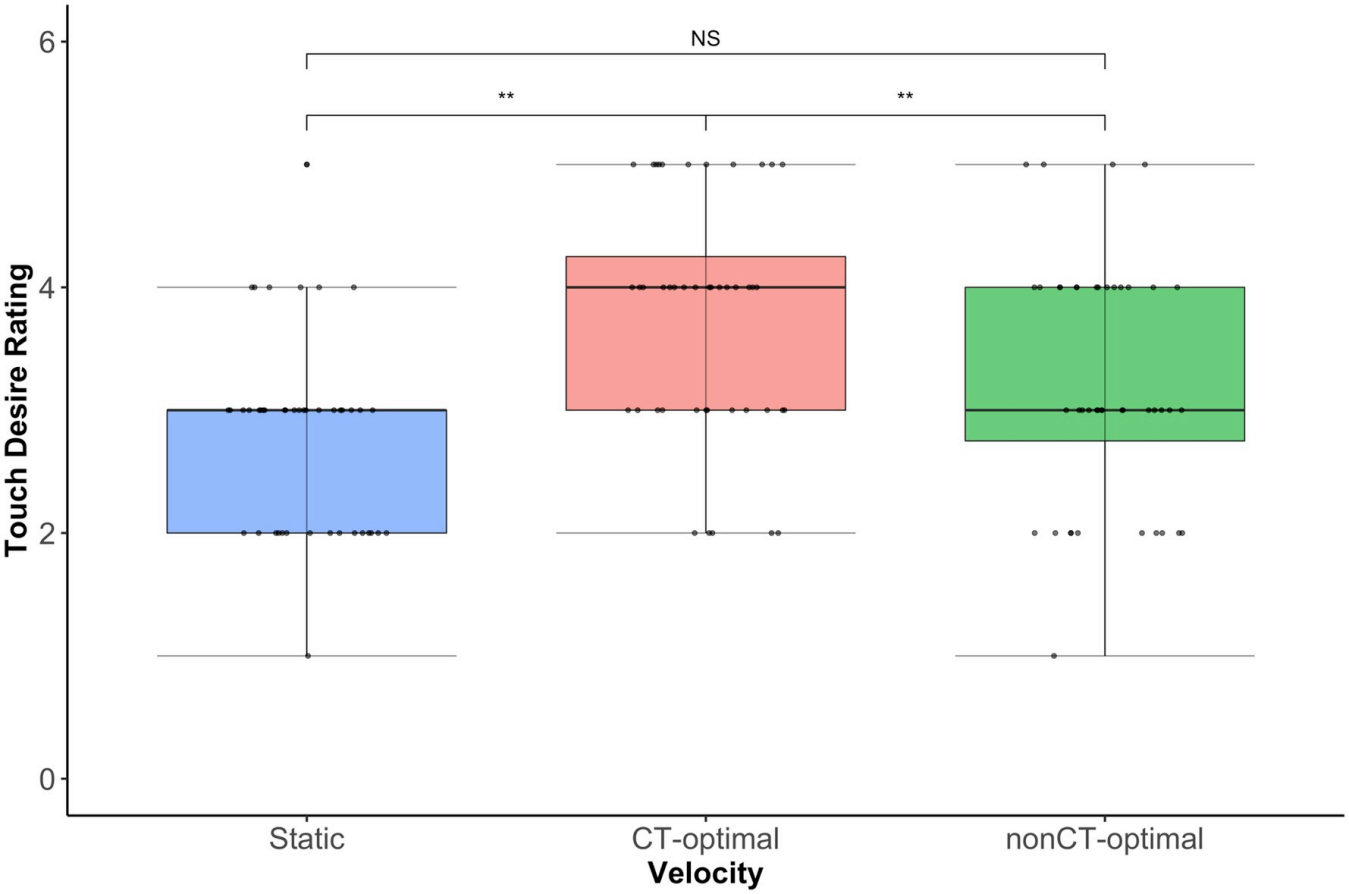
Box and whisker plot showing median pleasantness ratings (thick black line) for touch at the 3 stroking velocities for question 2. Dots represent individual participant ratings. As per question 1, there is a significant main effect of velocity (p < 0.001), but no effect of location, or location x velocity interaction. Wilcoxon Signed Ranks tests confirmed that the CT optimal velocity, slow stroking touch was rated significantly more positively than either static or non-CT optimal velocity touch, **ps<0.002.

For this question too, a significant main effect of velocity was identified (Wald χ^2^(2) = 30.96, *p* < 0.001) with Wilcoxon Signed Ranks tests again confirming CT optimal (~3cm/s) touch was rated significantly more positively than the other two velocities (*ps* < 0.002). Ratings of static and fast (~30cm/s) touch did not differ significantly (*p* = 0.14). For this question too, there was no significant of location (Wald χ^2^(4) = 7.72, *p* = 0.10), nor was there as significant location by velocity interaction (Wald χ^2^(8) = 12.95, *p* = 0.11). See Fig 3.

Thus, whether considering self or other, while children, like adults, rated the CT optimal (~3cm/s) touch as significantly more pleasant than either static or fast (~30cm/s) touch, their ratings did not vary across body sites.

### Relationship between responses to question 1 and 2

Spearman’s correlations confirmed that, as previously reported with adults [43], responses to the self and other questions were significantly correlated (static r_s_ = .34, *p* = 0.01, CT r_s_ = .55, *p* < .001, non-CT r_s_ = .57, *p* < .001).

## 4. Discussion

Consistent with our hypothesis, primary school age children rated the vicarious observation of slowly moving touch, optimal for activating CTs, as significantly more pleasant than static or faster, non-CT optimal touch. This finding is in line with previous studies in adults showing that seen-touch produces the same, velocity dependent affective responses as felt-touch [14, 18, 19, 43]. However, contrary to our prediction, children’s rating patterns do not vary according to the skin site being touched. This contrasts with two previous studies using these same video stimuli with adults, where touch on the back was rated higher than touch on more proximal arm and palm sites [19, 43]. Furthermore, unlike adults, children’s ratings of touch to the glabrous skin of the palm showed the same relationship between velocity and perceived pleasantness as on the other, hairy skin sites shown. In contrast, we have previously found that adults rate vicariously experienced, static touch on the palm equally pleasant to CT-optimal touch [19, 43]. Taken together, these findings show that, as with adults, children use velocity as a cue to determine the affective value of social touch. This is consistent with psychophysical, behavioral and neuroimaging studies showing the CT system is functional from birth and that children differentiate the affective qualities of CT optimal and non-CT optimal touch [23, 24, 32, 33]. This is also consistent with studies of vicarious pain which report affective resonance to dynamic depictions of painful touch, which, like our stimuli, lacked a broader social context [46].

The lack of topographical differentiation in the children’s ratings could be explained by several factors. On the one hand, children may not yet have had sufficient touch exposure and experience to differentiate affective values across skin sites. That could be because static touch to the palm, a hand on the shoulder, or a back rub are more typical of adult-to-adult interactions than the adult-child or child-child interactions, which the children are presumably drawing on to make their affective responses. Perceptions of touch are typically context dependent; how pleasant a given tactile interaction is reported to be varies both with who is doing the touching and where on the body the touch occurs [47]. Our stimuli excluded all social context from the clips shown and the touch occurs only at body sites where people rate touch positively in a range of social contexts [47, 48]. However, it could be that the topographical differentiations shown in adult ratings rely on top-down cognitive processes, drawing on a context which isn’t explicitly provided in the videos, while children’s ratings reflect a purely bottom-up affective resonance. If that is the case, it remains to be determined whether this difference is experience dependent or whether children lack the top-down cognitive empathic abilities necessary for the previously reported anatomical distinctions to emerge [9]. Future work could systematically test how contextual features, such as the age and gender of the actors in the social interaction, influence affective ratings. Alternatively, the inherently limited variance produced with the child-friendly rating scale we used here may have meant we lacked the sensitivity to differentiate between skin sites [45]. However, we think this is unlikely as in adults we only used a 7-point Likert scale and here children’s ratings did vary by velocity and were not at ceiling or floor for any clip presented.

Here, as with our previous study, we did not see any difference in the self-versus other focused questions we posed. This is consistent with previous studies with patient’s lacking c-fibres, whose vicarious ratings of moving touch mapped directly on to their ratings of directly experienced touch, providing strong evidence that affective resonance is indeed grounded in the viewer’s own perceptual experience [18]. It is also important to note that while here we would not necessarily expect the children’s experiences to differ strongly from others they know, in future studies the two questions may be useful in probing how trait and state factors modify vicarious ratings. For example, a number of studies have reported that neural responses to both experienced and seen touch vary in relation to several personality traits [49–51]. Furthermore, tactile sensitivities are commonly reported in children and adults with developmental disorders and autism spectrum conditions (ASC) [45, 52]. Indeed, fMRI data reveals blunted neural responses to affective touch in children and adolescents with ASC, in comparison to typically developing controls [53]. Thus, it would be interesting to determine whether these groups recognize that their affective tactile experiences are atypical to those of family and friends. A limitation of our study design may be that our self and other questions directly follow each other, which could prime participants to simply enter the same response again. We chose this design to try to avoid participants getting confused by the question they are answering at a given point. However, in future perhaps a blocked design, where participants rate all of the videos twice, once in relation to themselves and once in relation to a specific other, in a counterbalanced order, may improve sensitivity of this measure.

Given somatotopic organization within the posterior insula has been reported in the processing of both painful and pleasant tactile stimuli [54–56], we have previously proposed that the anatomical differentiation of adult’s vicariously experienced affective touch ratings reflect the hypothesized anatomical distribution of CTs [43]. However, given the absence of this differentiation in the current study, perhaps they rather reflect adult’s top-down, cognitive evaluation of the video stimuli. Future studies using fMRI may facilitate the testing of these alternative possibilities. While responses in posterior insula cortex to dynamic social, but not nonsocial, touch vary according to velocity [14, 18], activity here does not correlate with participant ratings of touch pleasantness. However, responses in posterior superior temporal sulcus and orbitofrontal cortex have previously been found to correlate with the subjective value of sensory stimuli, including affective touch [57–59]. Furthermore, activity in primary somatosensory, but not posterior insula, cortex has been found to vary according to the visual context in which a caress is experienced [60]. Thus, determining where in the brain these anatomically differentiated vicarious touch ratings are represented would give insight into the underlying sensory and cognitive processes.
